# The Tentorium Cerebelli: A Comprehensive Review Including Its Anatomy, Embryology, and Surgical Techniques

**DOI:** 10.7759/cureus.3079

**Published:** 2018-07-31

**Authors:** Rabjot Rai, Joe Iwanaga, Gaffar Shokouhi, Rod J Oskouian, R. Shane Tubbs

**Affiliations:** 1 Anatomy, St. George's University School of Medicine, St. George's, GRD; 2 Medical Education and Simulation, Seattle Science Foundation, Seattle, USA; 3 Neurosciences Research Center, Tabriz, IRN; 4 Neurosurgery, Swedish Neuroscience Institute, Seattle, USA; 5 Neurosurgery, Seattle Science Foundation, Seattle, USA

**Keywords:** tentorium cerebelli, tentorial notch, incisura, dural sinus, embryology

## Abstract

The tentorium cerebelli functions as a partition, dispelling the burden of weight from supratentorial structures upon inferior brain matter. Clinicians and neurosurgeons, when assessing pathological findings, should have knowledge regarding the tentorium cerebelli anatomy. This work of literature is a comprehensive review of the tentorium cerebelli, including its anatomy, embryology, and clinical and surgical implications. The evolutionary pattern demonstrates sequential stages to higher mammalian lineage. An understanding of the complexity of the neurovascular structures and the anatomy of the tentorium cerebelli is crucial for surgical procedures by neurosurgeons.

## Introduction and background

**Figure 1:**
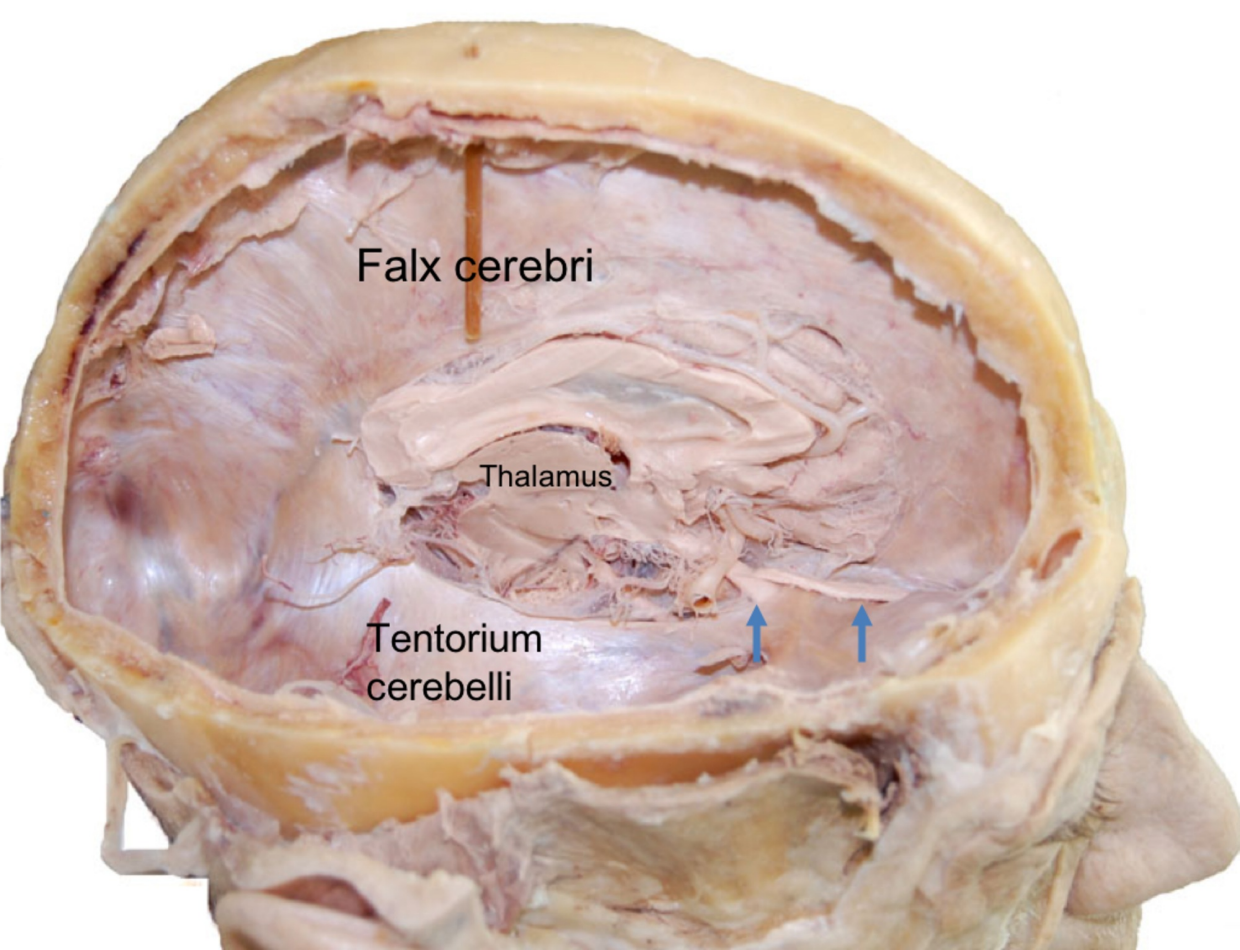
Right lateral view of the cranium after hemicraniectomy and removal of right cerebral hemisphere in a cadaver. Note the tentorium cerebelli and its relationship to the medial brain structures. Also, note the olfactory tract (right arrow) and optic nerve (left arrow).

**Figure 2:**
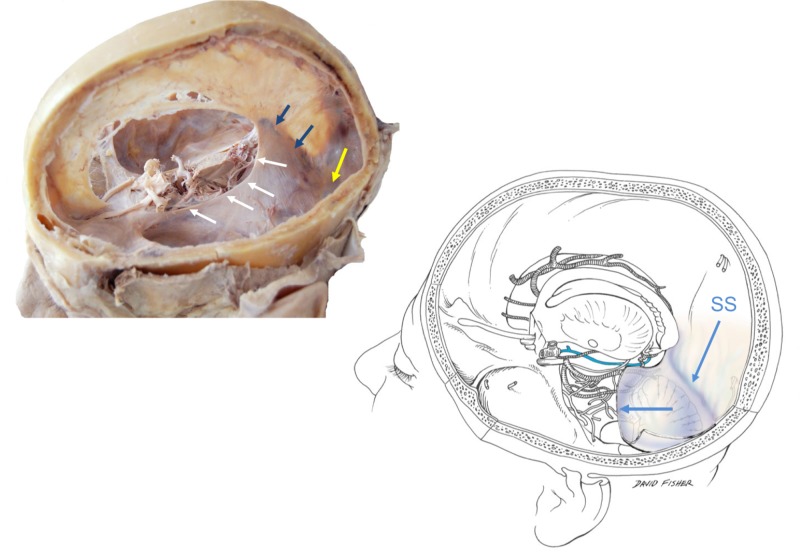
Left: the tentorial incisura (white arrows), straight sinus (blue arrows) and transverse sinus (yellow arrow); Right, schematic drawing of left hemicranium noting the numerous neurovascular structures just medial to the tentorial incisura e.g., basal vein of Rosenthal (blue). The anterior half of the tentorium cerebelli is cut away at the blue arrow. Also note the straight sinus (SS).

**Figure 3:**
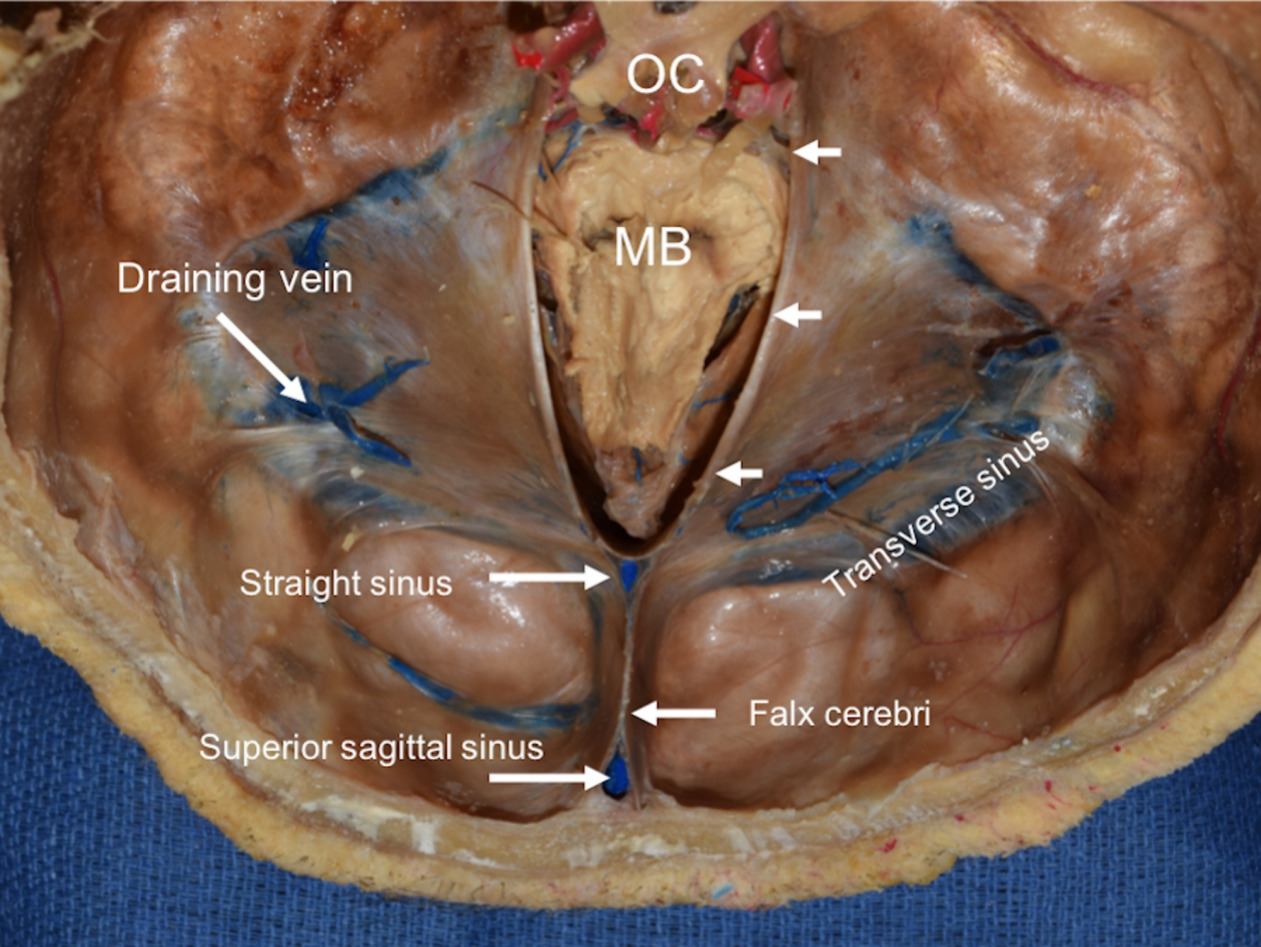
Superior view of a cadaveric tentorium cerebelli with brain superior to the brainstem removed. Note the midbrain (MB) and the optic chiasm (OC). White arrows point to the right tentorial incisura.

Three layers make up the meninges: the dura, arachnoid, and pia mater. The dura is also known as the thick meninx, or pachymeninx, while the arachnoid and pia mater are known as the thin meninx or leptomeninges [1]. An extension of the dura includes the dural reflections, which consist of four distinguishable dura folds, including the falx cerebri, tentorium cerebelli, falx cerebelli, and diaphragma sellae [2]. This review paper will look comprehensively at the tent-shaped structure forming the roof of the posterior cranial fossa, the tentorium cerebelli (Figures 1-3) [3]. The tent shape of the tentorium cerebelli helps maintain the anatomy of the brain by providing protection against the pressure caused by the heavier upper part of the brain [4-5]. If the tentorium cerebellum or falx cerebri were severed, sagging of the brain would take place [4]. However, the presence of the tentorium cerebelli can cause trouble during times of cranial swelling or displacement by space-occupying lesions [5].

## Review

Anatomy

**Figure 4:**
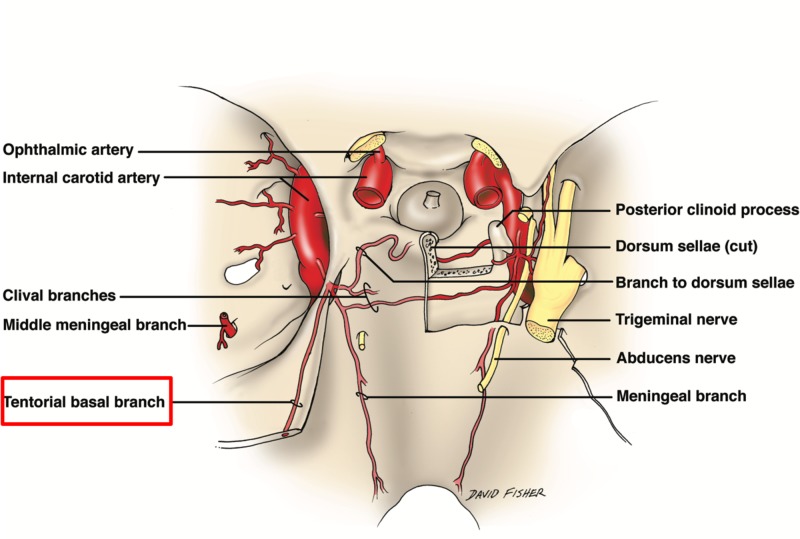
Schematic drawing of the anterior extensions of the tentorium cerebelli and the anterior petroclinoid and posterior petroclinoid folds, which make up the lateral and posterior borders of the oculomotor trigone.

**Figure 5:**
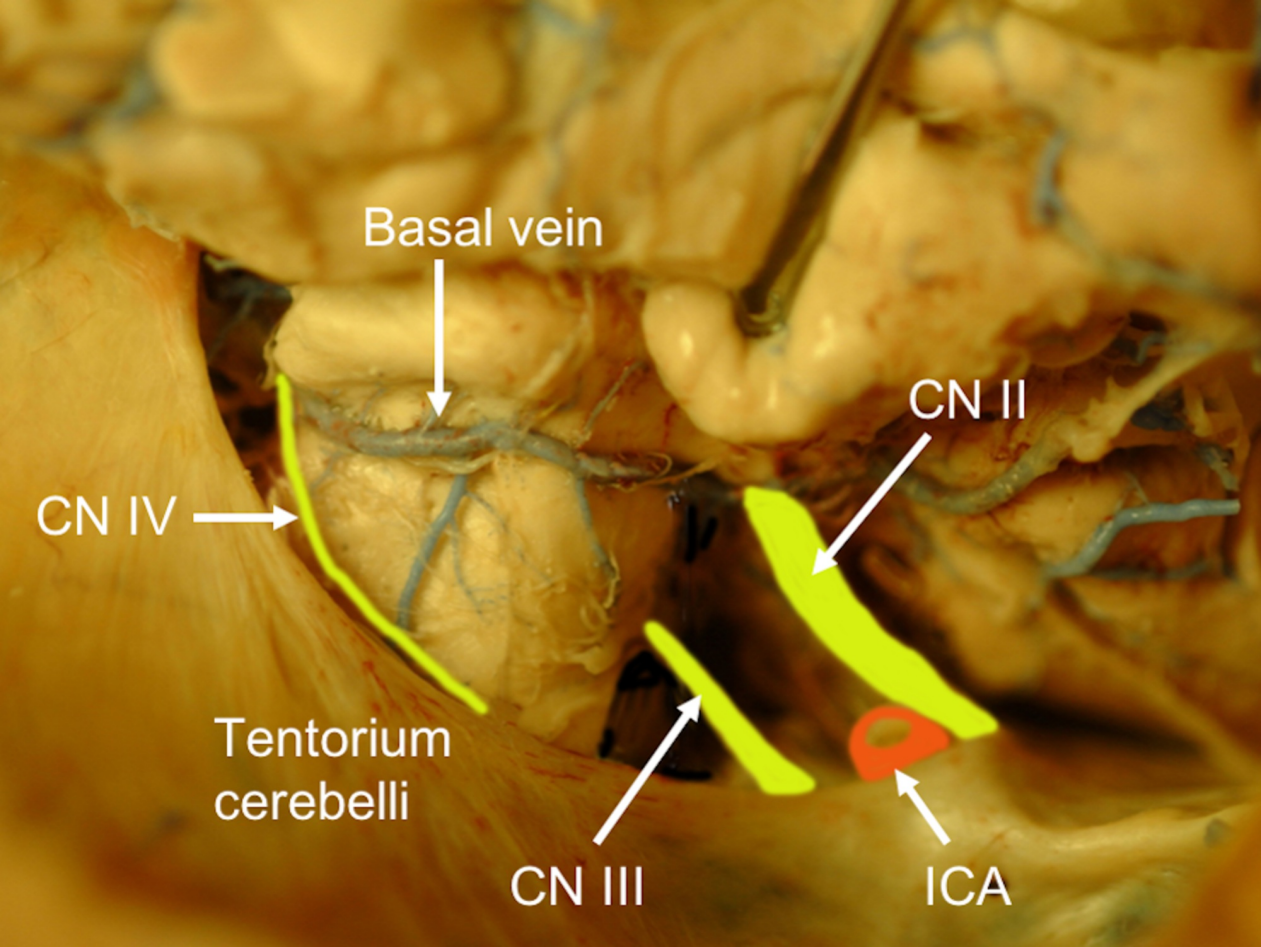
Right subtemporal view of the tentorium cerebelli and structures along its medial border such as the trochlear nerve. Anteriorly, note the internal carotid artery (transected) (ICA) for reference.

**Figure 6:**
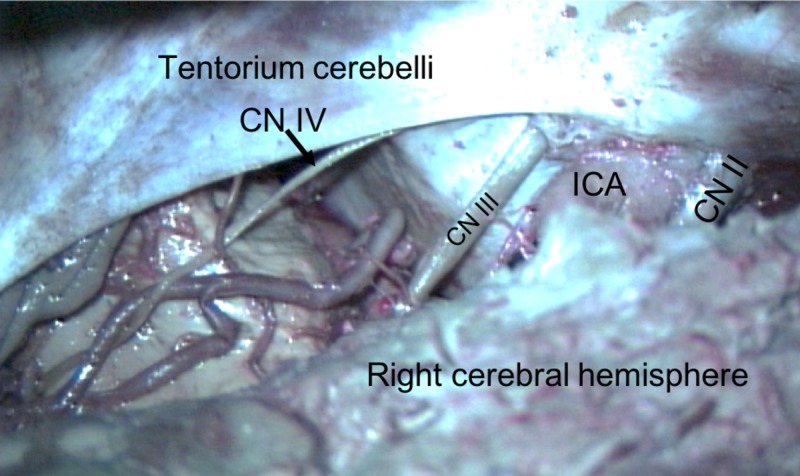
Superior view of the left tentorium cerebelli and its incisura. Note, the left cerebral hemisphere has been removed.

**Figure 7:**
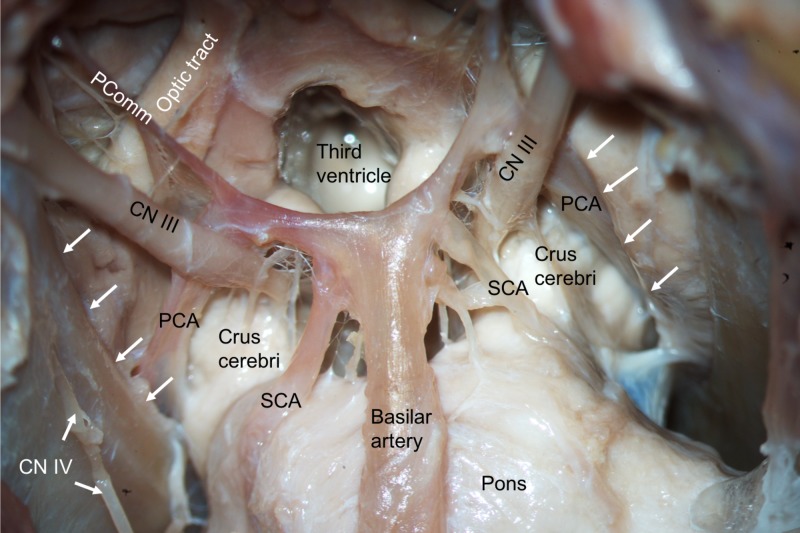
Inferior view of the tentorial incisura (white arrows) following a skull base approach. Note the posterior cerebral (PCA), superior cereberllar (SCA), and posterior communicating arteries (PComm).

The tentorium cerebelli, the second-largest dural reflection, is a crescent-shaped dura fold that extends over the posterior cranial fossa, separating the occipital and temporal cerebral hemisphere from the cerebellum and infratentorial brainstem [1,6]. The tentorial cerebelli is a distinguishing landmark and divides the cranial cavity into the supratentorial and infratentorial spaces [1]. This dural reflection has a free and fixed margin. The fixed margins of the tentorium cerebelli are attached to the superior borders of the petrous part of the temporal bone, known as the posterior clinoid process via the anterior and posterior petroclinoid folds (Figure 4) and along the transverse sinuses grooves on the occipital bone posteriorly. The free margin is located at the anterior edge and forms a U-shape termed the tentorial notch or the incisura tentoria (Figures 5-7). The tentorial notch allows for the presence of a gap, which lodges the midbrain [1,6]. The midbrain inhabits the anterior portion of the incisura and the posterior half is occupied by the superior vermis or the splenium of the corpus collosum. The remainder of the area is composed of the cerebrospinal fluid, blood vessels, and nerves within the perimesencephalic and superior vermis cisterns [3]. The free margin runs anteriorly, crosses the attached borders, and adheres to the anterior clinoid process bilaterally; this forms the lateral part of the cavernous sinus. At the location of the border crossing, cranial nerves III and IV pass through toward the lateral wall of the cavernous sinus [6]. The trigeminal nerve and trigeminal ganglion emerge between the recess formed by the apex of the petrous bone and the bottom layer of the tentorium pouched anteriorly beneath the superior petrosal sinus [1,6]. Above the free margin sits the hippocampal gyrus and the posterior cerebral artery; this anatomical finding is clinically important in cases involving transtentorial (uncal) herniations [7]. Lastly, the falx cerebri and falx cerebelli, which are also dural reflections, are attached to the tentorium cerebelli superiorly and inferiorly, respectively (Figures 1-3) [6].

Comparative anatomy

Klintworth’s (1968) study of the comparative anatomy found the tentorium cerebelli absent in amphibians, fish, and reptiles but present in birds and mammals [4]. The morphology of structure varied among different species, for instance, the tentorium was found as a separated bilateral delicate partition, unjoined at the midline, and dividing only the lateral portion of the cerebrum and cerebellum in bats, pigs, gerbils, hamsters, opossums, rats, and mice. While in other mammals (18 species mentioned), including cats, dogs, humans, rhesus monkeys, minks, and goats), the bilateral folds fused together at the midline give rise to a crescentic partition posterior to the brain stem, separating the posteroinferior portions of the cerebral hemisphere from the cerebellum. In these species, the posterior falx cerebri and, occasionally, a falx cerebelli adhered with the tentorium cerebelli. In these animals only, where the tentorium cerebelli and falx cerebri united in the median plane posteriorly, a straight sinus existed. Its length in relation to the tentorial notch varied among different species [4,8]. Klintworth [4] also found differences in the degree of tentorial ossification amongst various mammals. In his study, he found that in adult cats, the entire tentorium cerebelli was completely ossified. But, in dogs, dolphins, minks, porpoises, and wallabies, the ossification was limited to the posteromedial portion of the tentorium. According to Klintworth [4], the property of tentorial ossification provides an evolutionary pattern for mammalian lines. It reflects the retention of the osteogenic potentialities, which occur within the ectomeninx during the development of the neurocranium.

Anatomical variations

Brain stem hemorrhages are vascular lesions, which may lead to a mass effect and increased cranial pressure, giving rise to a herniation of the brain stem through the tentorial notch [4]. It was suggested by Corsellis [9] that the variation in tentorial notch size and shape might affect the pattern of herniation. Corsellis [9] reported these variations of the tentorium cerebelli, stating cases where it fit tightly around the brainstem to extreme cases where the hippocampal gyri were exposed. Although variations based on brain swelling or the cross-sectional area of the brain stem may be factors, Corsellis [9] had taken this into account and noted the changes to be strictly due to the size of the incisura. The size range of the incisura in the study concluded a range of 10 to 23 cm2, demonstrating that the largest noted measurement was twice the size of the smallest. Corsellis [9] stated that the tentorium is too strong and anchored to the cranium to be stretched once the completion of development occurs. Thus, the variability in size is due to the difference in the manifestation of normal development. However, it must be noted, these variations may give rise to a diverse pattern of herniation dependent on the size and shape of the tentorial notch. A study by Alder [10], examined the variation of tentorial notch apertures and its clinical significance. For instance, compared to short narrow notches, the long and wide notches allow for the greater exposure of the cerebellar tissue. As a result, this can explain the tendency for the transtentorial herniation of the cerebellar or cerebral parenchyma in the rostral or caudal directions, respectively. Similarly, variations in clinical presentation were found based on anatomical variants. A herniation of the hippocampus gyrus over the free margin of the tentorium cerebelli results in the compression of the oculomotor nerve. This direct pressure causes pupillary dilation. However, inconsistent findings of pupillary dilation in transtentorial herniation have been attributed to variations in oculomotor nerve length, as well as trajectory and degree of suspension in the tentorial hiatus [10].

Angulation

The tentorium cerebellum is a dural reflection that shields the posterior cranial fossa while being affixed to the cranial base and in union with the falx cerebri and falx cerebelli. As previously mentioned, this creates a partition of supratentorial and infratentorial regions. By creating this structural division, it allows the infratentorial region to be independent of the weight-bearing load from the supratentorial [11]. Bull [8] mentions a study by Handmann (1906) concluding that the average weight of an adult brain was 1,355 g while Wertham and Wertham (1934) concluded the weight of the cerebellum to be 150 g. As a result, the supratentorial brains weighs approximately 1,200 g. Bull [8] defined this concept as an important function of the tentorium cerebelli throughout evolution, where the partition transfers the weight of the cerebral hemisphere outwards towards the lateral wall of the cranium. This results in the weight being pushed away from the inferiorly positioned foramen magnum [12]. The tentorium cerebelli and falx cerebri are taut structures in order to restrain brain motion and diminish deformation across the midline [13]. Evolution plays a significant part, as man and higher primates support their head above the cervical spine rather than in front, requiring a mechanism to dispel extra weight onto the brainstem [8].

The partition of the tentorium cerebelli emulates a “tent” shape (Figures 1,5). At the midline, the tentorium adheres to the posterior region of the falx cerebri, which lies elevated compared to the lateral and posterior attachments of the tentorium to the skull. The free margin of the tentorial notch is convex downward while the posterior slopes upwards. This highest point descends outward laterally to its attachment to the occipital and temporal bones [14].

Embryology

A mass of mesenchyme, known as the prechordal plate, located rostral to the impending notochord, is the first embryological sign of brain development at stage 8 (day 16). At stage 9 (day 20), the mesoderm of the head is developed from the lateral migration of cells from the prechordal plate. The prechordal plate continues to generate mesenchyme. At stage 11 (day 24), the pia mater is identified at the caudal aspect of the medulla oblongata in reference to occipital somites derived from the neural crest. By stage 12 (day 26), the pia mater advances to the level of the mesencephalic. At this point, there is no distinction between neural crests, as they become incorporated with cranial-nerve ganglia and head mesenchyme. At stage 13 (day 28), the mesenchyme from the prechordal plate arranges bilaterally, causing the formation of premandibular condensation. At this time, the cellular sheath of the notochord is apparent within the occipital area and develops caudally. Between stages 14 and 17, the development of the medial aspect of tentorium cerebelli begins. At stage 14 (day 32), the extension of the notochordal cellular sheath is seen into the mesencephalic flexure. The premandibular condensation becomes continuous alongside the notochordal cellular sheath, reaching the tip of the notochord and resuming into the medial aspect of the future tentorium cerebelli. The development of the medial part of the tentorium cerebelli is predominantly leptomeningeal as the beginning of the formation is largely due to the involvement of the notochordal cellular sheath. In stage 17 (day 41), the dural limiting layer, where the pori durales for cranial nerves III, IV, V, and XII appear, begins formation within the basal areas. This is also where mesenchymal condensation for the formation of the future chondrocranium is forming. The dural limiting layer, lateral to the diencephalon, forms the rostrolateral portion of the tentorium cerebelli. During stage 19 (day 48), the leptomeningeal layer seen internal to the limiting layer is looser while the pachymeninx, where the future dural sinus will be located, is denser. The medial aspect of the tentorium cerebelli is condensed to fibrous tissue, and by stage 21 (day 52), it extends from the sella turcica to the mammillary body, however, this medial part starts to become thinner. By stage 23 (day 57), the lateral portions of the tentorium extend close to the ridge of the mesencephalon. The rostrolateral portion meets the caudolateral at the medial part, which begins to disintegrate. The line of union of the two tentorial leaves at the site of the future tentorial notch. The medial part disappears, and the rostrolateral and caudolateral portions form the definitive tentorial cerebelli [15].

Histology

The tentorium cerebelli is visible under the microscope in a crown-rump length of 20 mm. By eight weeks, the embryos tentorium crown-rump length is approximately 30 mm and can be grossly viewed as delicate bilateral folds. By three months, with a crown-rump of 55 mm, the union of the bilateral folds posteriorly give rise to the development of the straight sinus. As the fetus grows, the tentorium and tentorial notch increase in size progressively. Microscopically, at the stage of the six to eight weeks embryo, the tentorium examination comprised of loosely packed areolar connective tissue of spindle or stellate cells is bordered by a layer of flattened mesothelial cells superiorly and inferiorly. As the tentorium continues ontogenesis, further histogenesis identifies bundles of collagen at the central core amassing, which evolve into a dense fibrous membrane [16].

Arterial vasculature supply

**Figure 8:**
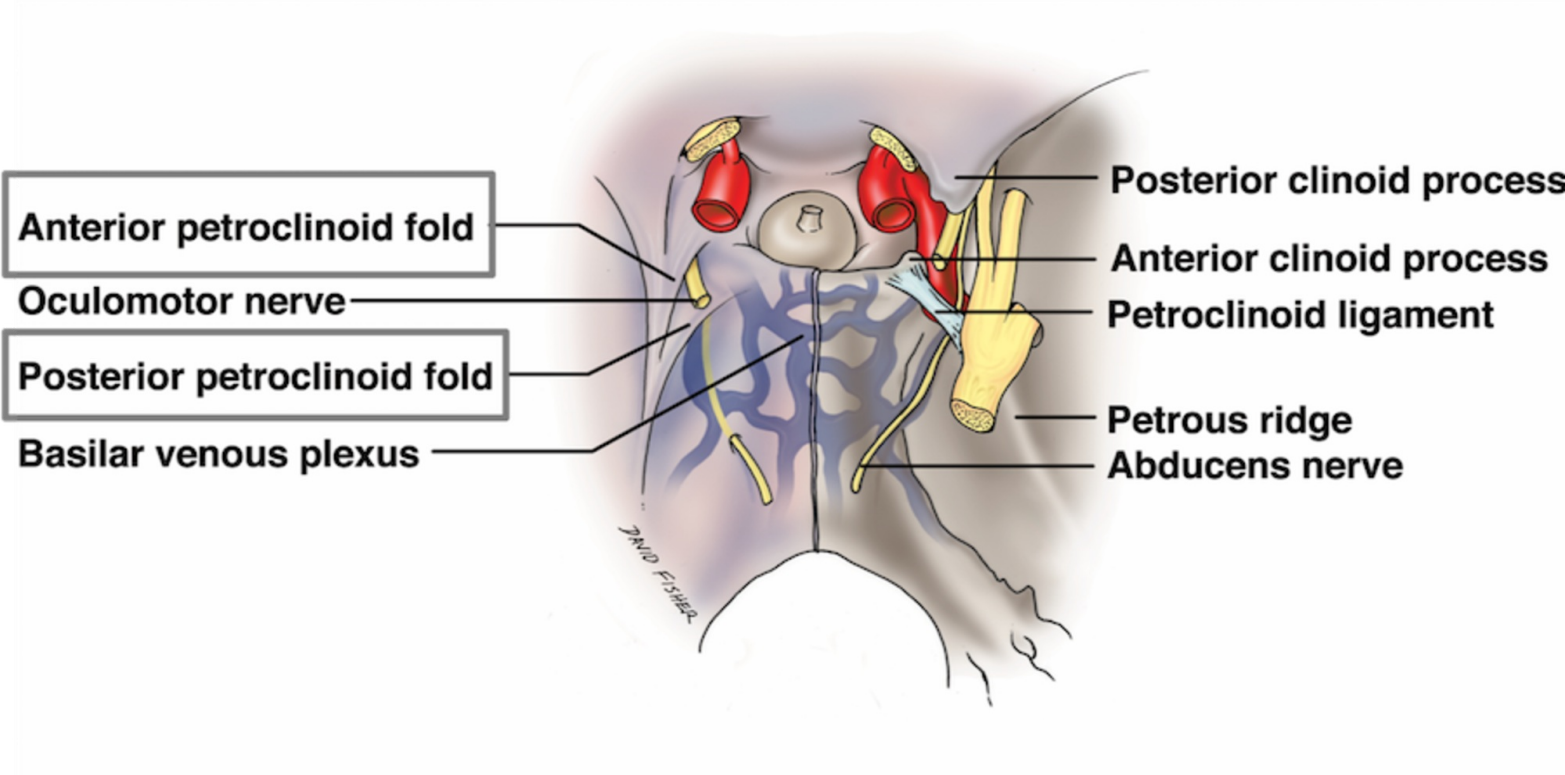
Small vessels of the skull base noting the tentorial branch to the free edge of the tentorium from the meningohypophyseal trunk of the cavernous internal carotid artery.

The various anastomotic networks between adjacent arterial regions further complicate the complexity of the vascular supply of the tentorium cerebelli [2]. The meningeal branches of the external carotid, internal carotid, posterior cerebral, and vertebral arteries are all involved in the blood supply to the dura of the posterior fossa, including the tentorium cerebelli. A small meningohypophyseal trunk arising from the intracavernous portion of the internal carotid artery branches into two arteries, the tentorial and dorsal meningeal (Figure 8). The tentorial artery, also known as the artery of Bernasconi and Cassinari, exits from the cavernous sinus and travels between the dural foldings of the tentorium. The tentorial artery branches posterolaterally and becomes parallel to the free margin, one branching off and supplying the tentorium as it heads towards the straight sinus [17-18]. The other branching laterally provides vasculature to the lateral portion of the tentorium [18]. The posterior cerebral artery gives rise to small infratentorial branches, known as the artery of Davidoff and Schechter, which travel around the brain stem and under the free margin toward the tentorium apex, where they supply the medial aspect of the tentorium cerebelli. The internal carotid artery and branches of the external carotid artery provide vascular supply to the petrosal insertion of the tentorium [19]. The vertebral artery branches into the posterior meningeal artery, where its supratentorial branches supply the inferior falx cerebri, adjacent dura, and adjacent tentorium cerebelli [20].

Dural venous sinuses associated with the tentorium cerebelli

Straight Sinus

The straight sinus is formed by the merger of the inferior sagittal sinus with the great cerebral vein of Galen. The straight sinus is at the junction between the tentorium cerebelli and the falx cerebri. The sinus drains into the confluence of the sinuses or transverse sinus [21]. The straight sinus is angled downward in a rostrocaudal direction when viewed vertically [22].

Transverse Sinus

The transverse sinus emerges laterally from the confluence of the sinus while adhering to the tentorium cerebelli posteriorly. The right transverse sinus is usually larger compared to the left, which is primarily caused by drainage from the superior sagittal sinus directing to the right. As a result, the right transverse sinus, right sigmoid sinus, and right internal jugular vein drain venous blood from the superficial areas of the brain, while the left transverse sinus, left sigmoid sinus, and left internal jugular vein drain blood from deeper regions via the internal cerebral vessels and the basal and great veins [21].

Superior Petrosal Sinus

The superior petrosal sinus travels in the attached part of the tentorium cerebelli [21-22]. The superior petrosal sinus drains the cavernous sinus and extends toward the transverse sinus.

Tentorial Sinuses

Tentorial sinuses collect various supra and infratentorial bridging veins and are bilateral but more or less asymmetrical venous channels. The medial group is formed by the coalescence of the veins from the superior surface of the cerebellum, and the lateral group is commonly formed by the convergence of veins arising from the basal and lateral surface of the temporal and occipital lobes. The medial group arises about 1.5 to 2.5 cm from the midline and about 1.0 to 2.5 cm from the transverse sinus. The lateral sinuses are often found 3.0 to 4.0 cm from the midline, 2-10 mm from the transverse sinus, and 1.0 cm from the petrous ridge.

Innervation

The recurrent meningeal branches of V1 (ophthalmic division of the trigeminal nerve), also known as the nervus tentorii of Arnold, innervates the tentorium cerebelli. The distribution of the nervus tentorii investigated by Lee et al. [23] identified four morphological divisions. The pattern of type 1 had the highest prevalence in the study, which concluded that the fibers extend to the straight and transverse sinus. Type 2 exhibited the fibers extending only to the transverse sinus and lateral convexity. Type 3 projected the fibers medially towards only the straight sinus and posterior portion of the falx cerebri. Lastly, type 4 exhibited the nerve fibers terminating within the tentorium cerebelli. The study conducted by Lee et al. [23] found type 1 to have the highest prevalence (the incidence rate of type 1-4 were 71.2%, 21.2%, 3.8%, and 3.8%, respectively) from 52 samples of the tentorium cerebelli from 29 cadavers (both sides from 23 cadavers, and one side from six cadavers). The distribution patterns of the nervus tentorii were asymmetrical when comparing the left and right sides of the tentorium cerebelli.

The posterior projections of these tentorial nerves form a plexus within the tentorium cerebelli and are predominate at the superior wall of the transverse sinus and the posterior half of the straight sinus. Lee et al. [23] found innervation to be the most scarce at the tentorial notch and the anterior half of the straight sinus. This provided clinical significance so as to avoid retraction at the posterior aspect of the falx cerebri and tentorium cerebelli. Manipulation in this area was shown to cause hemodynamic fluctuations, including hypotension, bradycardia, arrhythmia, asystole, or apnea (known as the trigeminocardiac reflex) by eliciting neural signals stimulated by the sensory endings of the trigeminal nerve through the trigeminal ganglion to the sensory nucleus of the trigeminal nerve in the brainstem [23-24].

Imaging

The use of contrast-enhanced computed tomography (CT) is able to visualize the tentorium in 99% of cases but can be difficult to recognize on non-contrasted images. The inclination in which the image was taken contributes to the varied configuration for which the tentorium is recognized. It can be viewed as four types: (1) Gothic arch — a plane of the incisura showing the full contour of the tentorium from the anterior clinoid process to the apex of the tentorial notch, (2) V configuration — a plane of the torcular showing with the tentorium traveling medially, (3) Diverging bands — a plane below the torcular showing where the tentorium departs posterolaterally towards its lateral attachments, and (4) M configuration — a plane through the torcular.

With pathological findings, the tentorial bands may be more prominent on contrast CT scans, including in arteriovenous malformation, venous sinus thrombosis, and causes of tentorial hypervascularity [3]. A majority of the intracranial space-occupying lesions can be identified through cranial CT alone without the use of neuroradiological procedures. The CT scans are able to localize the mass lesions, however, secondary effects are less visible [25]. As mentioned, visualization of the tentorium on non-contrast images is difficult, however, when pathological states occur, the visualization of the tentorium becomes evident. Pathological findings, such as tentorial calcification, subarachnoid hemorrhages, tentorial hypervascularity, and juxtatentorial brain atrophy are able to demarcate the tentorium on non-contrast CT images [3].

The thin nature of the tentorium cerebelli causes its contrast imaging to be low compared to surrounding tissues. With gadolinium-enhanced T1-weighted magnetic resonance imaging (MRI), the visibility of the tentorium is improved, however, a limiting factor is accessibility to the contrast [13]. The use of contrast-enhanced cranial CT and MRI are the best methods for the imaging of the tentorium cerebelli.

Clinical and surgical nuances

Brain Herniation

Brain herniation is a term to describe the displacement of brain matter through a rigid opening of the skull or dura mater, such as through the dural reflections of the tentorium cerebelli and falx cerebri. The pathological states of space-occupying lesions, such as brain tumors, intracranial hemorrhage, or cerebral edema, can cause the brain to herniate when sufficient compensation is not achievable. The most common brain herniation includes uncal, central, subfalcine, upward, and tonsillar. From these, the uncal, upward, and central are associated with the tentorium cerebellum, being the rigid opening for the protrusion of brain tissue. Herniations are a life-threatening syndrome requiring immediate attention. Although surgical intervention is crucial, stabilizing measures can be taken place including hyperventilation, osmotic therapy, and elevating the head of the bed in order to acutely decrease intracranial pressure and improve cerebral perfusion pressure. It is important to note that the mainstay of treatment is surgical intervention. It is essential in order to decrease intracranial pressure via the removal of the mass effect, the removal of cerebral structures (lobectomy), or decompressive craniotomy in order to create another opening for the brain to displace, thus reducing the downward and lateral compression [7]. Herniation might be caused as a result of intracranial hemorrhages from the subdural or epidural hematoma; the standard of care includes burr-hole exploration and emergency craniotomy [26].

Tentorial Neoplasms

Due to the vascular supply and neural structures surrounding the tentorium cerebelli, surgical intervention to debulk tumors within the tentorium can be difficult [27]. Bassiouni et al. [27] investigated the microsurgical approach to tentorial meningioma and found a prevalence of 3%-6% amongst all intracranial meningioma. The best approach to prevent reoccurrence involved tumor removal with a resection of dural involvement and diseased bone if cancer cells invaded. Surgical manipulation in the tentorium cerebelli raises concerns about the possible resection of the dural venous sinus. Due to the high vascularity of the region, Bassiouni et al. [27] used a more conservative approach when tumors invade a patent sinus wall, coagulate residual tumor on the sinus wall, or resect the outer dural layer of the infiltrated sinus wall. This option leaves the remnants of the tumor behind. On the other hand, there is an overall consensus that a completely occluded and non-patent sinus wall can be removed safely.

*Surgical Approaches* 

The surgical technique for approaching a tumor varies based on the location of the lesion in relation to the tentorium cerebelli. Ideally, a lateral tentorial lesion is approached both above and below the tentorium in order to expose margins beyond the tumor. The central core of the lesion is removed, and then the surrounding tumor along the occipital and cerebellum are dissected. Tumors located medially to the tentorium at the tentorial notch are best approached via an occipital transtentorial, compared to a supracerebellar infratentorial approach, allowing for the tumor to be devascularized prior to resection via sectioning around the tentorium [15]. The tentorium can be split to offer a larger surgical corridor. The main complication in this approach is an injury to the striate cortex affecting vision [27]. The most common location for tumors is at the petrosal apex, and these are best approached either via the subtemporal or retromastoid approaches depending on whether the tumor is supratentorially or infratentorially located, respectively. The subtemporal approach allows for a better visualization of the anterior and lateral midbrain, pons, posterior cerebral, posterior communicating, and superior cerebellar arteries and oculomotor and trochlear nerves [15]. This route may require temporal lobe resection, thus, a venogram to assess the anatomy should be performed prior to lobectomy [27]. The retromastoid approach, on the other hand, allows for the safer exposure of the lower cranial nerves [15]. Approaches to the tentorial notch, especially when this structure is transected, must appreciate the trochlear nerve and avoid it as anteriorly, it will pierce the tentorium to enter the cavernous sinus and this point can be variable. Along the attached margin of the tentorium to the petrous ridge, one must take care not to injure the superior petrosal sinus, especially as it crosses the trigeminal nerve at the opening of Meckel’s cave.

## Conclusions

As the higher primates evolved from quadrupedalism to bipedalism with erect posture, the tentorium cerebellum displays successive stages of evolution through mammalian lines. As the weight shifts from the front end to above the cervical spine, the tentorium cerebelli adds strength to the midline to aid in supporting the extra weight. The difference in the shape and size of the tentorium throughout development may have a clinical effect during herniation, resulting in different brain tissues herniating through the rigid tentorial incisura depending on anatomical variant. The understanding of the complexity of the neurovascular structures around the tentorium cerebelli is crucial for neurosurgeons when operating. The higher neuronal structures in the posterior tentorium cerebelli should be avoided during surgical procedures. Prior to operating, a venogram to depict the venous and dural structure is indicated for individual variation. Knowledge of the anatomy will support clinical diagnosis and guide management for the neurosurgeon.
